# Lowering Ionic Strength Improves the Sensitivity of Microtubule Gliding Assay Based Molecular Detection

**DOI:** 10.1021/acs.nanolett.5c01188

**Published:** 2025-05-08

**Authors:** Eugene Christo V R, Esther Charlotte Sophia Kloth, Filippo Nisini, Cordula Reuther, Stefan Diez

**Affiliations:** 1 B CUBE – Center for Molecular Bioengineering, TUD Dresden University of Technology, 01307 Dresden, Germany; 2 Cluster of Excellence Physics of Life, TUD Dresden University of Technology, 01062, Dresden, Germany; 3 Max Planck Institute for Molecular Cell Biology and Genetics, Dresden 01307, Germany

**Keywords:** Point-of-care diagnostics, Molecular detection, Assay sensitivity, Gliding motility assay, Kinesin, Microtubule

## Abstract

Microtubule gliding assays provide a unique mechanism for molecular detection in which binding of analytes to the microtubule lattice reduces the microtubule gliding speed. The reduction in the gliding speed correlates with the density of the bound analytes, enabling its quantification. Although promising, this technique is still in the proof-of-concept stage. Improving the sensitivity and limit of detection of the assay could make the technique comparable to that of advanced molecular detection methods. This study demonstrates that reducing the ionic strength of the buffer increases the sensitivity of the assay by enhancing the interactions between kinesin and microtubules. When using a low ionic strength buffer (BRB10) compared with a standard buffer (BRB80), we observed a more pronounced reduction in microtubule gliding speed in the presence of analytes, improving the detection limit. Therefore, this approach offers a simple and scalable way to improve the sensitivity of motor-based detection assays.

## Graphical abstract

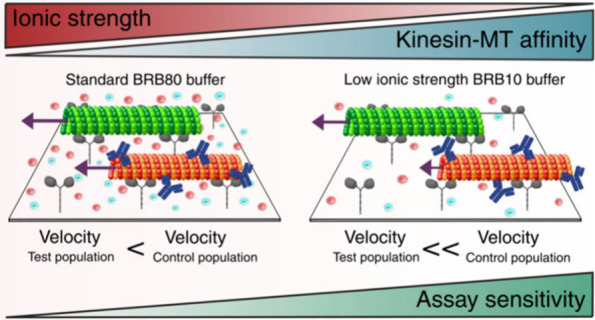

Biomolecular motors, such as kinesins, are a family of ATP-dependent motor proteins crucial for force generation and transport of cargo within the cell.^1,2^ They are engineered by nature to function efficiently at the nanoscale.^3^ Among them, Kinesin-1 motors (henceforth referred to as kinesin) have been studied extensively.^4^ These motors move along microtubules, which are composed of α- and β-tubulin dimers arranged into a hollow, polarized cylinderal structure.^5^ Each kinesin motor possesses two motor heads that bind to the microtubule lattice through electrostatic and hydrophobic interactions. By harnessing energy from ATP hydrolysis, kinesin undergoes conformational changes, enabling it to “walk” along the microtubule in a highly coordinated fashion. The unique properties of kinesin-microtubule motility, such as energy efficiency and directional movement, have been exploited *in vitro* using gliding motility assays.

In a gliding motility assay, microtubules are driven by a carpet of kinesin motors immobilized on a substrate surface. Recent advances in biocompatible nanofabrication allows directed movement of microtubules on patterned surfaces, enabling spatial control over gliding microtubules, making them suitable for engineering purposes.^6–10^ Kinesin driven microtubules have been exploited in various nanotechnological applications ranging from biocomputing,^11^ cargo transport,^12–14^ molecular sorting,^15–17^ and surface imaging^18^ to biomolecule detection.^19–21^ Among these applications, the detection of biomolecules based on kinesin driven microtubules has emerged as a promising approach that can be used at the point-of-care.

The molecular detection method utilizes a gliding motility scheme, where the target analytes bind to the microtubule lattice, usually via antibody–antigen interactions. When analytes are bound to the microtubule surface, they act as obstacles that impede the kinesin motors that propel the microtubules, until the motor either bypasses the obstacle or detaches from the microtubule lattice. This phenomenon, known as the roadblock effect, results in a reduction of microtubule gliding speed. Since the degree of speed reduction correlates with the density of bound analytes, the concentration of analytes in solution can be quantified from the microtubule gliding speeds.^22^ This approach can be adapted to detect a wide range of analytes by functionalizing the microtubule lattice with receptor molecules, such as full antibodies, Fab fragments, or short peptides. Previous studies have demonstrated its applicability to analytes of varying sizes and shapes, for example, the detection of creatine kinase,^23^ streptavidin, neutravidin, avidin, and antibodies^20,22,23^ as well as for the direct detection of tau protein binding to the microtubule lattice.^24,25^ Furthermore, the compatibility of the assay with complex biological fluids, such as whole blood and cell lysates, has been demonstrated.^26^ Despite their potential, molecular motor-based detection systems remain in the proof-of-concept stage. While the assay offers several advantages over conventional techniques like ELISA including rapid detection, ease of use, and potential for point-of-care applications, the sensitivity of the assay is currently comparable to existing point-of-care methods such as lateral flow assays.^27,28^

Improving the sensitivity and detection limit would expand the usage of roadblock assays in diagnostics that require precise quantification and detection of low analyte concentrations. The slope of the decrease in velocity with respect to the analyte concentration can be used as a measure of the sensitivity of these assays. An increase in the sensitivity would result in a more pronounced decrease in the microtubule gliding speed when the analytes bind to the microtubule lattice. The detection limit can be measured as the minimum analyte concentration that causes a measurable decrease in gliding speed. Enhancing the sensitivity and detection limit could make the motor-based detection method compete with advanced state-of-the-art detection methods like ELISA and bio barcoding assay.^29–31^ Despite a strong need to explore and develop strategies that could increase the sensitivity of these assays, to date, there have been only scarce efforts.

In this work, we tested if enhancing the affinity between kinesin and microtubules could amplify the roadblock effect, as the motors facing an obstacle would remain bound to the microtubule longer, slowing the microtubule gliding speed more profoundly. Given that kinesin-microtubule binding is dominated by electrostatic interaction,^32^ we expected that modifying buffer conditions, i.e. reducing the salt concentration, will enhance the affinity between kinesin and microtubules.

We thus investigated how the reduced ionic strength affects kinesin-microtubule interactions and assay sensitivity. We performed gliding motility assays and single-molecule stepping assays in standard BRB80 buffer and reduced ionic strength buffer. In gliding motility assays, we observed the movement of microtubules propelled by kinesin motors, and in stepping assays we tracked the motion of single GFP-labeled kinesin motors along immobilized microtubules. This provided insights into how the ionic strengths of the buffers used in this study affect kinesin dynamics in a collective and at the single-molecule level. Under reduced ionic strength conditions, we observed an increase in kinesin-microtubule affinity evident from the slower gliding speed in the microtubule gliding assay along with reduction in stepping velocity and increase in run length and residence time of kinesin motors in stepping assays as compared to standard BRB80 buffer. We then evaluated how ionic strength impacts the sensitivity of the roadblock assay. We observed a more pronounced reduction in the microtubule gliding speed in the presence of analytes under low ionic strength conditions.

To investigate the impact of low ionic strength conditions on microtubule motility, we performed gliding motility assays comparing standard BRB80 buffer (ionic strength: 184 mM; see Methods for calculations) with low ionic strength BRB10 buffer (ionic strength: 28.85 mM) (Figure 1A). The BRB10 buffer had a 6-fold reduction in ionic strength relative to the BRB80 buffer, providing a substantially altered electrostatic environment for kinesin-microtubule interactions. Microtubule gliding motility was monitored over a period of 30 minutes in both buffers, which were supplemented with 10 μM taxol, 2 mM MgATP and antifade agents. The microtubules exhibited consistent gliding under both buffer conditions throughout the observed period, although significant differences in gliding speed were observed between the two buffers (Figure 1B). The microtubule gliding speeds were calculated from microscopy images as frame-to-frame velocities using MATLAB (Supplementary Figure S1). In BRB80 buffer, the gliding speed was 594 ± 46 nm/s (median ± interquartile range, *n* = 477 microtubules) whereas the speed decreased to 497 ± 49 nm/s in BRB10 buffer (*n* = 373 microtubules). To assess the reversibility of this slowdown effect, we switched buffer conditions within the same flow channel, sequentially from BRB80 to BRB10 and then back to BRB80. Under initial BRB80 conditions, microtubule gliding velocity was 606 ± 44 nm/s (*n* = 274 microtubules). Upon replacing BRB80 with BRB10, the velocity decreased to 494 ± 38 nm/s (*n* = 424 microtubules). And after switching back to BRB80, the velocity increased again to 622 ± 39 nm/s (*n* = 368 microtubules) (Figure 1C). These findings confirm that the reduction in microtubule gliding speed under low ionic strength conditions (BRB10) is completely reversible, and kinesin motors remain fully functional.

**Figure 1:**
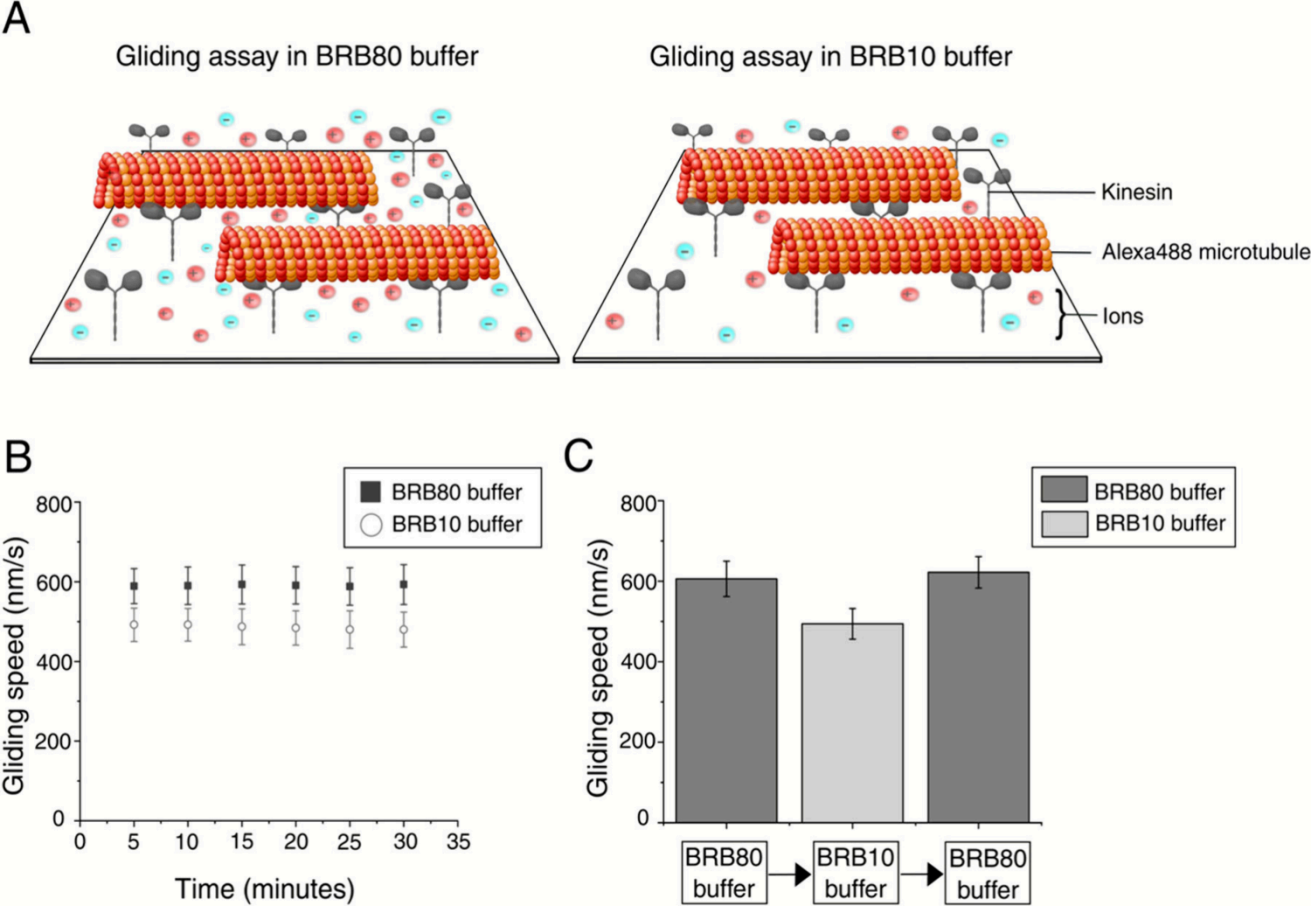
Impact of low ionic strength on microtubule gliding speed. (A) Schematic representation of the experimental setup for microtubule gliding motility assays conducted in standard BRB80 (high ionic strength) and BRB10 (low ionic strength) buffers. Alexa488-labeled microtubules, shown in orange, are propelled by surface-bound kinesin motors, depicted in gray. (B) Plot showing the microtubule gliding speed in BRB80 and BRB10 buffers over a 30 min period. Stable microtubule gliding was observed. An overall reduction in gliding speeds was observed in the BRB10 buffer. Markers and error bars represent the median and interquartile range. (C) Bar graph comparing the gliding speeds of microtubules when the buffers are alternated within the same channel between BRB80 and BRB10 buffers. A reduction in microtubule gliding speed was observed in BRB10, which increased again when BRB10 was replaced with BRB80 motility buffer, demonstrating the reversibility of this effect. Markers and error bars represent the median and interquartile range. All experiments were conducted at room temperature (22.95 ± 0.04 °C, mean ± SD). The differences in gliding speeds between the two different buffers were found to be statistically significant on a 95% confidence level (Wilcoxon rank-sum test, *p* < 0.001).

To confirm pH stability, particularly under low ionic strength conditions, we monitored the pH of both BRB80 and BRB10 buffers over a 30 min period. The buffers contained antifade agents (20 mM d-glucose, 20 μg/mL glucose oxidase, 10 μg/mL catalase, and 10 mM DTT) along with 2 mM MgATP, and 10 μM taxol to replicate experimental conditions. The pH remained sufficiently stable in both buffer conditions throughout the duration of the experiment (Supplementary Figure S2).

To quantify the effect of reduced ionic strength on the kinesin-microtubule interaction at single molecule level, we performed kinesin steppings assay under both buffer conditions. Alexa647-labeled microtubules were immobilized on the surface using anti-β-tubulin antibodies, and the motion of individual GFP-labeled kinesin-1 motors was tracked with total internal reflection fluorescence (TIRF) microscopy (Figure 2A). The experiments were performed at a controlled temperature of 28 °C. The temperature was maintained by using an objective heater. Our findings revealed a significant increase in the run length of kinesin motors in low ionic strength conditions, as shown in the space-time kymographs and cumulative distribution frequency plots (Figure 2B,C). In BRB80 buffer, the average run length was 951 ± 188 nm (mean ± standard deviation, *n* = 114), whereas it increased to 2260 ± 382 nm (*n* = 116) in BRB10 buffer. Additionally, the interaction time of kinesin motors with microtubules was increased under low ionic strength conditions (Figure 2D). In BRB80 buffer, the average interaction time was 1.0 ± 0.2 s (*n* = 114), which increased to 3.4 ± 0.6 s (*n* = 116) in BRB10 buffer. These results align with the enhanced run lengths observed in BRB10 and indicate a stronger kinesin-microtubule interaction under reduced ionic strength. Consistent with our gliding motility assays, the stepping velocity of the kinesin motors was reduced under low ionic strength conditions. In BRB80 buffer, the stepping velocity was 904 ± 46 nm/s, whereas in BRB10, it decreased to 660 ± 21 nm/s (Figure 2E,F). These findings are consistent with previous reports^33^ and strongly support the notion that the reduced ionic strength buffer used in this study enhances the kinesin-microtubule interaction, as indicated by the increased interaction times and run lengths. While the attachment rate (*k*_on_) is influenced by the motor protein concentration in solution, the detachment rate (*k*_off_) depends solely on the motor-filament interaction.^34^ Therefore, the detachment rate, which is an indicator of kinesin microtubule affinity, can be calculated as the inverse of interaction time. In BRB80 buffer, *k*_off_ was 1.0 ± 0.2 s^–1^, whereas in BRB10 buffer, the *k*_off_ decreased three-fold to 0.29 ± 0.05 s^–1^. Together, the results from both the gliding motility and single-molecule stepping assays demonstrate that reducing the ionic strength increases the affinity between kinesin motors and microtubules.

**Figure 2:**
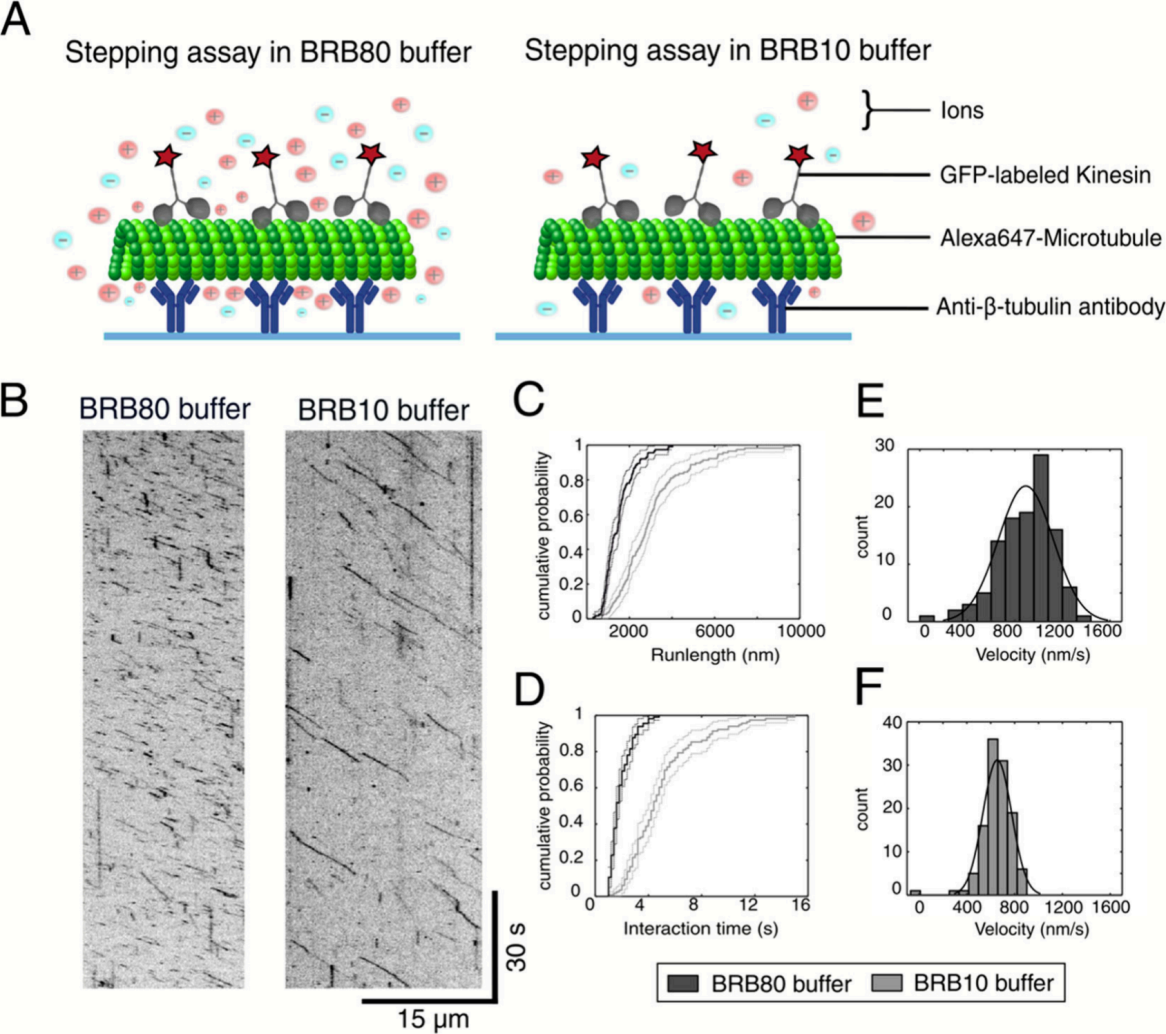
Impact of low ionic strength on single molecule kinesin stepping. (A) Schematic representation of the experimental setup for kinesin stepping assays conducted in standard BRB80 (high ionic strength) and BRB10 (low ionic strength) buffers. GFP-labeled kinesin motors (shown in grey) move along Alexa647-labeled microtubules (shown in green), which are immobilized on the surface via anti-β-tubulin antibodies (shown in blue). (B) Kymograph illustrating kinesin movement along microtubules in BRB80 and BRB10 buffers. The traces are visibly longer in BRB10, indicating an increased run length under low ionic strength conditions. (C) Cumulative distribution plot of kinesin run length in BRB80 and BRB10 buffers, confirming the increased run length seen in the kymographs. (D) Cumulative distribution plot of kinesin–microtubule interaction times in BRB80 and BRB10 buffers. An increase in interaction time is observed in BRB10. Error bars represent the 95% confidence interval. Error bars represent the 95% confidence interval. (E) Velocity of kinesin motors in BRB80 buffer. (F) Velocity of kinesin motors in BRB10 buffer. Experiments were conducted at a controlled temperature of 28 °C using an objective heater. Differences in interaction time, run length, and velocity between buffers were statistically significant on a 95% confidence level (Student’s t-test, *p* < 0.001).

Next, we investigated how the buffer’s ionic strength impacts the sensitivity of the roadblock assay. For this experiment, we used two populations of microtubules: The control group consisted of Alexa647-labeled microtubules and the test group of Alexa488-labeled microtubules with different concentrations of anti-Alexa488 antibodies, which acted as roadblocks (Figure 3A). To ensure that the number of antibodies bound to the microtubules was the same in different buffer conditions, which would allow a direct comparison of the extent of slowdown, anti-Alexa488 antibodies were always bound to microtubules in BRB80 buffer and incubated for 2 min in a flow channel already containing kinesin, microtubules, and motility solution. Subsequently unbound antibodies were removed by flushing with a BRB80 buffer. The gliding speeds of these microtubules were monitored in both BRB10 and BRB80 buffers, at varying concentrations of anti-Alexa488 antibodies, which acted as roadblocks. For each antibody concentration, the microtubule gliding speed in the two buffers BRB80 and BRB10 was then measured successively in the same flow channel. As expected, an increase in anti-Alexa488 antibody concentration led to a reduction in the gliding speed of the test microtubules in both buffer conditions (Supplementary Figure S3). However, the reduction in gliding speed was more pronounced in the low ionic strength BRB10 buffer compared to BRB80. To quantify this effect, relative gliding speeds were calculated as the ratio of the gliding speed of Alexa488-labeled test microtubules to Alexa647-labeled control microtubules, expressed as a percentage (median ± interquartile range; see Materials and Methods). The comparison of relative gliding speeds of the microtubules across varying anti-Alexa488 antibody concentrations under both buffer conditions is illustrated in Figure 3B. At very low anti-Alexa488 antibody concentrations (1 nM), the difference in relative gliding speeds was small between the two buffers, with values of 99.8 ± 11.0% in BRB80 and 96 ± 10% in BRB10. While the slowdown observed in BRB80 at this concentration was not significant, BRB10 showed a significant reduction in test microtubule gliding speed. In BRB80 buffer conditions, a measurable reduction in the gliding speed of the test population was only observed for 2 nM anti-Alexa488 antibody concentration (relative gliding speed: 92 ± 15%). This indicates that the limit of detection improved by a factor of 2 in low ionic strength environments. To confirm that this effect was not simply due to time delays between measurements, we replaced the BRB10 buffer with BRB80 in the same channel following imaging at a 1 nM anti-Alexa488 antibody concentration. The gliding speeds increased, and no reduction in microtubule gliding speed was observed between the two microtubule populations, supporting that the observed sensitivity increase was specific to the low ionic strength environment (Supplementary Figure S4).

**Figure 3:**
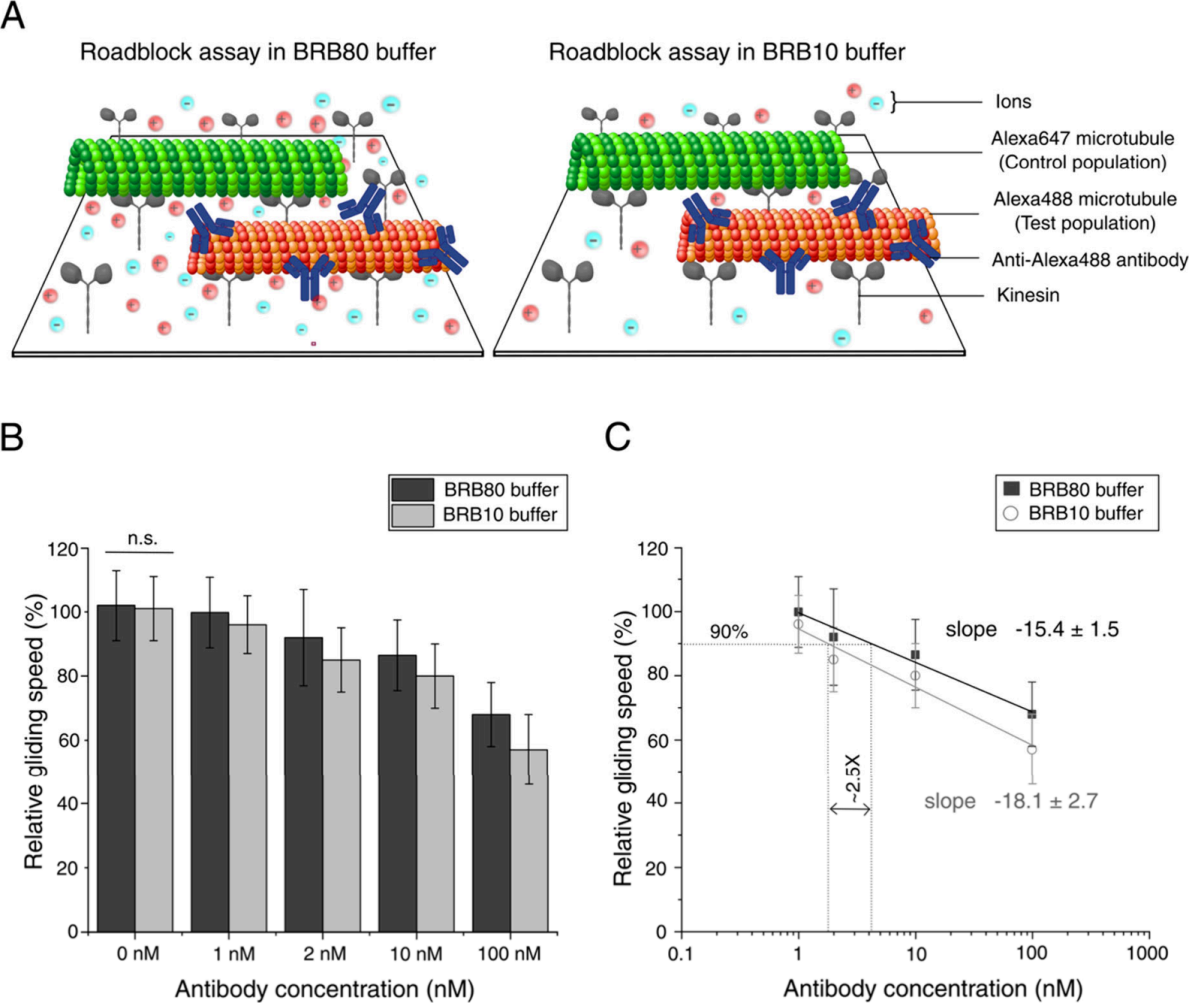
Roadblock assay comparing the effect of BRB80 and BRB10 buffers under varying anti-Alexa488 antibody concentrations. (A) Schematic illustration of the experimental setup. The control population consists of Alexa647-labeled microtubules (shown in green), while the test population consists of Alexa488-labeled microtubules (shown in orange), which bind to anti-Alexa488 antibodies (shown in blue). Antibody binding slows the test population of microtubules, creating a roadblock effect. Microtubule gliding speed was monitored in both BRB80 (left) and BRB10 (right) buffers at varying anti-Alexa488 antibody concentrations. (B) Bar plot comparing the relative gliding speed of microtubules at different anti-Alexa488 antibody concentrations in BRB80 and BRB10 buffers. A more pronounced decrease in gliding speed is observed in BRB10, indicating enhanced sensitivity under low ionic strength conditions. Markers and error bars represent the median and interquartile range. The relative gliding speeds for the different buffer conditions were significantly different on a 95% confidence level for antibody concentrations of 1 to 100 nM (Wilcoxon rank-sum test, *p* < 0.001) and not significantly different for 0 nM (n.s.). (C) Scatter plot showing the relationship between relative gliding speed and anti-Alexa488 antibody concentration (nM). Linear regression analysis reveals a steeper slope for BRB10 compared to BRB80, reflecting increased sensitivity in the lower ionic strength buffer. Markers and error bars represent the median and interquartile range.

At higher anti-Alexa488 antibody concentrations, the enhanced sensitivity of BRB10 was more pronounced. For example, at 100 nM anti-Alexa488 antibody concentration, the relative gliding speed in BRB80 was 68 ± 10%, while in BRB10 it was further reduced to 57 ± 11%. A linear correlation was observed between relative gliding speeds and the anti-Alexa488 antibody concentration in the log scale, with a steeper slope in BRB10 buffer (Figure 3C). The slope of the linear fit was measured to be 15.4 ± 1.3% in BRB80 buffer and 18.1 ± 2.7% in BRB10 buffer. This slope (in units of % relative speed per log­[concentration]) was used as an empirical comparative metric of sensitivity and was not intended to represent a mechanistic binding model. Since the increase in slope under low ionic strength conditions was a measure of increased sensitivity, this result emphasizes the ability of low ionic strength conditions to detect more subtle differences in roadblock density.

To validate that the observed enhancement in assay sensitivity under low ionic strength conditions was actually due to stronger kinesin-microtubule interactions and not just a result of slower microtubule gliding speeds, we reduced the ATP concentration in the BRB80 buffer from 2 to 0.15 mM to match the microtubule gliding speeds with low ionic strength conditions, while maintaining the final MgCl_2_ concentration at 3 mM, consistent with standard BRB80 buffer. We then performed roadblock experiments using 100 nM anti-Alexa488 antibody. The gliding speed of the control microtubules was matched with 491 ± 44 nm/s (median ± interquartile range, *n* = 327 microtubules) in the modified low ATP BRB80 buffer and 482 ± 41 nm/s in BRB10 buffer (*n* = 432 microtubules) quite well. We observed a significantly greater slowdown under low ionic strength conditions compared to BRB80 buffer, even with matched gliding speeds. The relative gliding speed of microtubules in BRB80 buffer was 67 ± 10% (median ± interquartile range), and in modified low ATP BRB80 buffer the relative gliding speed was 65 ± 12%, while the relative gliding speed in low ionic strength BRB10 buffer was 57 ± 9% (Figure 4A), indicating that the increased sensitivity was not a mere consequence of slower gliding speeds in low ionic strength conditions.

**Figure 4:**
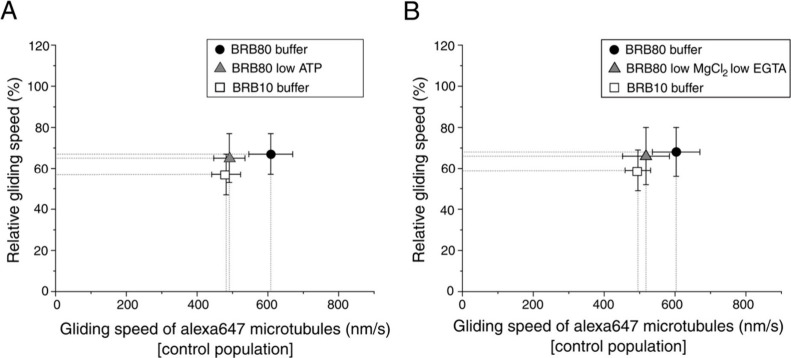
Roadblock assay comparing the performance of standard BRB80 buffer with modified BRB80 buffer and BRB10 buffer. (A) Comparison of the effect of a modified BRB80 buffer with reduced ATP concentration (0.15 mM ATP) on the roadblock assay at 100 nM anti-Alexa488 antibody concentration, relative to standard BRB80 and BRB10 buffers. The *x*-axis represents the microtubule gliding speed of the control population in nm/s (Alexa647-labeled microtubules), while the *y*-axis shows the relative gliding speed (%) of the test population. Markers and error bars represent the median and interquartile range. (B) Comparison of the effect of a modified BRB80 buffer with 0.25 mM EGTA, 0.25 mM MgCl_2_, and 2 mM Mg-ATP on the roadblock assay at 100 nM anti-Alexa488 antibody concentration, relative to standard BRB80 and BRB10 buffers. The *x*-axis represents the microtubule gliding speed of the control population (Alexa647-labeled microtubules), while the *y*-axis shows the relative gliding speed (%) of the test population. Markers and error bars represent the median and interquartile range. Statistical significance of the results on a 95% confidence level was confirmed using the Wilcoxon rank-sum test (*p* < 0.001).

We further investigated the impact of differences in buffer composition beyond the PIPES concentration. Specifically, while BRB80 contains 1 mM MgCl_2_ and 1 mM EGTA, BRB10 has 0.25 mM of each. To investigate the role of differences in magnesium concentration (co-factor for ATP hydrolysis^35^), we performed roadblock assay experiments with 100 nM anti-Alexa488 antibodies and compared BRB10 buffer to both standard BRB80 buffer and modified BRB80 buffer (Mg^2+^ and EGTA concentration matched to BRB10 buffer). The results demonstrated a significantly greater relative slowdown under low ionic strength conditions in BRB10, with a relative gliding speed of 59 ± 10%, compared to 68 ± 12% in standard BRB80 and 66 ± 14% in modified BRB80, as shown in Figure 4B. These findings indicate that the improved sensitivity observed in low ionic strength conditions was not solely due to the reduced Mg^2+^ and EGTA concentrations in BRB10, highlighting the role of overall ionic strength in enhancing roadblock assay performance. To further evaluate this, we performed roadblock assays with an intermediate buffer (BRB20) using a 100 nM anti-Alexa488 antibody concentration. As shown in Supplementary Figure S5, relative gliding speeds decreased with decreasing ionic strength across BRB80, BRB20, and BRB10, indicating a consistent increase in assay sensitivity with lowering the ionic strength.

Our study demonstrates that reducing the ionic strength of the buffer significantly enhances the sensitivity of roadblock assays by strengthening the binding affinity between kinesin and microtubules. Under standard BRB80 buffer conditions, weaker electrostatic interactions result in a reduced affinity between kinesin and microtubules. In contrast, the low ionic strength BRB10 buffer promotes stronger interactions, leading to a more pronounced reduction in microtubule gliding speed in the presence of roadblocks (Figure 5). The reduction in ionic strength of the buffer effectively improves the detection limit of the assay two-folds under low ionic strength conditions. Although this study used antibodies as roadblocks to demonstrate improved sensitivity, the enhancement arises from increased kinesin–microtubule affinity; therefore, a similar improvement could be expected for other analytes that act as roadblocks. While this improvement may appear modest, this was the first study to demonstrate sensitivity enhancement in a gliding assay-based biosensor. Further improvements could be achieved by increasing kinesin–microtubule affinity, for example, by using alternative motors with intrinsically longer run lengths, i.e., stronger motor-microtubule affinities, or by engineering motor domains that retain even stronger binding under low ionic strength conditions.

**Figure 5:**
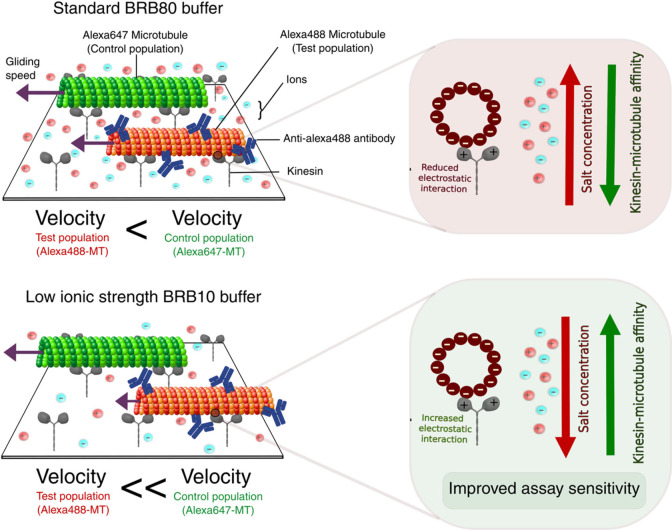
Enhanced sensitivity of the roadblock assay through increased kinesin–microtubule affinity at lower ionic strength. Schematic illustrating the principle that reducing ionic strength increases the sensitivity of the roadblock assay by enhancing kinesin-microtubule binding affinity. Top: At higher ionic strength (BRB80 buffer), the electrostatic interaction between kinesin and microtubules is reduced due to higher salt concentration, resulting in weaker kinesin–microtubule binding. This lowers the assay’s sensitivity to roadblocks; therefore, the relative gliding speed difference between control and test population is lesser. Bottom: At lower ionic strength (BRB10 buffer), decreased salt concentration strengthens electrostatic interactions between kinesin and microtubules, increasing kinesin–microtubule affinity. This enhanced binding prolongs kinesin interaction with microtubules, amplifying the roadblock effect and thereby improving assay sensitivity. Therefore, relative difference in gliding speed between control and test population is higher. The insets depict the relationship between salt concentration and kinesin–microtubule affinity, with stronger interactions observed at reduced ionic strength, leading to improved sensitivity in detecting analyte binding.

Moreover, reducing the ionic strength below BRB10 could be of interest. This was not feasible in our system because lowering ionic strength reduces buffering capacity, making it difficult to maintain a stable pH, particularly in fluorescence microscopy assays that rely on oxygen-scavenging antifade systems. Commonly used agents such as glucose oxidase generate acidic byproducts, leading to a gradual pH drop that must be compensated with increased buffer strength. As a potential solution, future studies could explore alternative antioxidant systems such as pyranose oxidase, which perform more effectively under low ionic strength condition.^36^

The sensitivity of the roadblock effect depends on several factors including the size, charge and distance of the roadblock from the microtubule lattice.^20^ Proteins as small as ∼15 kDa (e.g., mono-streptavidin) have been shown to cause measurable slowdown of gliding microtubules.^23^ suggesting a lower size threshold for direct detection. For smaller analytes, detection can still be achieved through secondary binding to larger molecules such as antibodies, Fab fragments, or aptamers, similar to amplification strategies in immunoassays, thus broadening the assay’s applicability.

In terms of binding density, the assay has been shown to detect up to ∼16 streptavidin roadblocks per micron of microtubule length when biotin is incorporated into the lattice.^22^ However, its sensitivity is constrained by the kinetics of binding of analytes to microtubules at very low concentrations. Addressing this challenge could enable more analyte binding to the microtubules. One potential solution is increasing the incubation time with analytes. While the lowest detectable antibody concentration in this study was 1 nM, the incubation time with microtubules was limited to just two minutes. Extending this incubation time could allow more roadblocks to bind to microtubules, further enhancing the assay sensitivity. Additionally, using high affinity antibodies or incubating antibodies in a low ionic strength buffer could strengthen the interactions, consequently increasing the roadblock density on microtubules and improving the detection limit, especially at low analyte concentrations.

Implementation of alternate readout methods could broaden the practical applications of this assay. For instance, performing gliding assays on the tip of an optical fiber allows estimation of microtubule gliding speeds based on the decay of fluorescence signal over time, as microtubules glide off the fiber tip.^23^ Such methods would remove the need for direct imaging, simplifying the implementation. Assuming a 10% reduction gliding speed as a detectable signal in such assays, our findings indicate a 2.5-fold improvement in detection limit while also improving sensitivity (Figure 3C).

While this study was conducted under controlled conditions, previous work has demonstrated the compatibility of gliding assays with complex biological media, including diluted whole blood, serum, and cell lysates.^26^ Our assay relies on relative gliding speed and includes an internal control population, which helps compensate for buffer- or sample-induced variability in absolute gliding speed. This design makes the assay robust even in less controlled environments. The extent to which the ionic strength can be lowered in such sample solutions will depend on the specific application and target analyte.

In summary, we show that reducing the ionic strength of the buffer improves the sensitivity and detection limit of the roadblock assay by increasing the affinity between kinesin and microtubules, establishing a mechanistic link between kinesin-microtubule affinity and detection sensitivity. Control experiments confirmed that this improvement is specifically due to enhanced kinesin-microtubule interactions, independent of microtubule gliding speed or changes in MgCl_2_ (co-factor) concentrations. These findings present a simple and scalable approach to increase the sensitivity of motor-based assays for point-of-care diagnostics requiring high sensitivity and improved detection limits.

## Supplementary Material



## Notes

Materials and methods; figures showing (i) microtubule tracking and speed analysis, (ii) pH stability of BRB80 and BRB10 buffers, (iii) roadblock effect under varying antibody concentrations in BRB80 and BRB10 buffer, (iv) buffer-switching experiments to indicate reversibility of increased sensitivity in low ionic strength conditions, and (v) ionic strength dependence of assay sensitivity (PDF)
