# Supplementary material for: Lowering Ionic Strength Improves the Sensitivity of Microtubule Gliding Assay Based Molecular Detection
Source: Nano Lett. 2025 May 8;25(20):8194–202. doi: 10.1021/acs.nanolett.5c01188 (PMC12100704; doi:10.1021/acs.nanolett.5c01188)
Supplement: Supplementary file 1 [file nl5c01188_si_001.pdf]

SUPPLEMENTARY INFORMATION

LOWERING IONIC STRENGTH IMPROVES THE SENSITIVITY OF MICROTUBULE GLIDING ASSAY BASED MOLECULAR DETECTION

Eugene Christo V R[1], Esther Charlotte Sophia Kloth[1], Filippo Nisini[1], Cordula Reuther[1], Stefan Diez[1,2,3]

[1] B CUBE – Center for Molecular Bioengineering, TUD Dresden University of Technology, 01307 Dresden, Germany

[2] Cluster of Excellence Physics of Life, TUD Dresden University of Technology, 01062 Dresden, Germany

[3] Max Planck Institute for Molecular Cell Biology and Genetics, 01307 Dresden, Germany

MATERIALS AND METHODS

Materials

All materials were purchased from Sigma Aldrich unless otherwise specified.

Buffers

This study utilized buffers with different concentration of PIPES:

BRB80 (standard buffer): 80 mM PIPES, 1 mM EGTA, 1 mM $MgCl_2$, adjusted to pH 6.9 with KOH.

BRB10 (low ionic strength buffer): 10 mM PIPES, 0.25 mM EGTA, 0.25 mM $MgCl_2$, adjusted to pH 6.9 with KOH.

BRB20 (used for flow cell preparation in kinesin stepping assays): 20 mM PIPES, 1 mM EGTA, 1 mM $MgCl_2$, adjusted to pH 6.9 with KOH.

The motility solutions contained 2 mM MgATP, 20 mM D-glucose, 20 µg/mL glucose oxidase, 10 µg/mL catalase, 10 mM DTT, and 10 µM taxol in either BRB80 or BRB10 buffer. The final $MgCl_2$ concentration was 3 mM in the BRB80 motility solution and 2.25 mM in the BRB10 motility solution.

Ionic Strength Calculations

The ionic strengths of the buffers used in this study were calculated using the formula, as described previously[1]. BRB80 buffer consists of 80 mM PIPES, 1 mM $MgCl_2$, 1 mM EGTA, and 2 mM MgATP, titrated to pH 6.9 with KOH. BRB10 buffer contains 10 mM PIPES, 0.25

mM $MgCl_2$, 0.25 mM EGTA, and 2 mM MgATP, also titrated to pH 6.9 with KOH. 2 mM MgATP solution was prepared with 2 mM $MgCl_2$ and 2 mM disodium ATP, and the resulting contributions to ionic strength were accounted for in the calculations. For BRB80, the dissociation of PIPES at pH 6.9 resulted in 33.6 mM $PIPES^{-1}$, 46.4 mM $PIPES^{-2}$, and 126.4 mM K^+, yielding an ionic strength contribution of 173 mM. In BRB10, the dissociation produced 4.2 mM $PIPES^{-1}$, 5.8 mM $PIPES^{-2}$, and 15.8 mM K^+, contributing an ionic strength of 21.6 mM. The contributions of Mg^{2+}, EGTA, and ATP were calculated by considering the free and bound species of each and their respective ionic strength effects. Additional contributions from 4 mM Na^+ (from disodium ATP) and 6 mM Cl^- (from $MgCl_2$ for BRB80 buffer and 4.5 mM Cl^- for BRB10 buffer) were also included. When combining the contributions from PIPES, Mg^{2+}, EGTA, ATP, Na^+, and Cl^-, the final estimated ionic strengths were determined to be 184 mM for BRB80 and 28.85 mM for BRB10.

pH Measurements

The pH of 400 μL solutions of BRB80 and BRB10 buffers was monitored in the presence of antifade agents (20 mM D-glucose, 20 μg/mL glucose oxidase, 10 μg/mL catalase, 10 mM DTT), along with 2 mM MgATP and 10 μM taxol. pH measurements were performed using a Mettler Toledo InLab Ultra-Micro-ISM pH probe, and the pH was measured every 5 minutes, over a 30-minute period.

Expression and Purification of Tubulin and Kinesin-1

Porcine tubulin was purified from porcine brain tissue following established protocols[2] and subsequently labeled with AlexaFluor 488 or AlexaFluor 647. Kinesin-1 from *Drosophila melanogaster* was expressed in insect cells and purified as described previously[3].

Microtubule Preparation

Alexa488- and Alexa647-labeled microtubules were polymerized in BRB80 buffer (80 mM PIPES, 1 mM EGTA, 1 mM $MgCl_2$, pH 6.9), supplemented with 5 mM $MgCl_2$, 1 mM MgGTP, and 5% DMSO. Polymerization was performed at 37 °C for one hour using a 4 mg/mL tubulin mixture. The microtubules were then stabilized with 10 mM Taxol in BRB80 buffer and stored at room temperature. All experiments were conducted within 24 hours of microtubule polymerization.

Gliding Motility Assays in BRB80 and BRB10 Buffers

Gliding motility assays were conducted in flow cells using the following protocol: A solution of 0.5 mg/mL casein in BRB80 was flowed into the cell and incubated for 5 minutes. Next, a 12.5 nM kinesin-1 solution (BRB80 supplemented with 0.2 mg/mL casein, 1 mM ATP, and 1 mM DTT) was introduced and incubated for 15 minutes. Then, motility solution containing microtubules (2 mM MgATP, 20 mM D-glucose, 20 μg/mL glucose oxidase, 10 μg/mL catalase, 10 mM DTT, and 10 μM taxol in BRB80) was then added and incubated for 1 minute. Excess microtubules were subsequently washed off with motility solution.
All the preparation steps until this point were performed in standard BRB80 buffer and were used consistently across all gliding and roadblock experiments. Once the flow cell was prepared and microtubules were loaded, the channels were sealed with vacuum grease, and microtubule gliding was observed under a microscope for 30 minutes.

For low ionic strength measurements, after removing excess microtubules with BRB80 motility buffer, the buffer was exchanged three times (using three times the flow cell volume) with BRB10 motility solution. The channels were then sealed, and gliding behavior was monitored for 30 minutes.
To ensure consistency across conditions, a single flow cell channel was used for each study condition, alternating between BRB80 and BRB10 within the same channel. For all gliding experiments, this approach ensured uniform conditions when comparing the effects of BRB10 with BRB80, with microtubules imaged for 5 minutes in each buffer. All the microtubule gliding experiments were conducted at room temperature (22.95 ± 0.04 °C), monitored by a temperature sensor on the stage of the microscope.
For buffer-switching experiments, after flow cell preparation and microtubule loading, the channel was first washed three times with BRB80 motility buffer (using three times the flow cell volume), and microtubules were imaged for 5 minutes. The buffer was then exchanged three times with BRB10 motility buffer in the same manner, and microtubules were again imaged for 5 minutes. Finally, the buffer was switched back to BRB80 with three exchanges, followed by a final 5-minute imaging session. This procedure allowed for direct observation of the effects of buffer ionic strength on gliding behavior, while minimizing variability from sample preparation.

Kinesin Stepping Assays in BRB80 and BRB10 Buffers

For stepping assays, cover slips were prepared in the following way. Firstly, they were cleaned with piranha solution (H_2O_2/H_2SO_4, 3:5) and then silanized with 0.05% dichlorodimethylsilane (DDS) in trichloroethylene. Channels were then made on the silanized glass cover slips and incubated with 0.5 μM monoclonal anti-β-tubulin antibody (Sigma Aldrich, clone SAP.4G5) in BRB20 for 5 minutes, followed by a 60-minute incubation with 1% Pluronic F-127 in BRB20. Microtubules in BRB80 were then introduced and incubated for 5 minutes. Single-molecule recordings were subsequently performed in BRB80 and BRB10 motility buffers, containing the same concentrations of reagents as previously described.
For kinesin motility experiments in BRB80 buffer, the motility buffer was supplemented with 40 nM truncated, GFP-labeled kinesin-1 constructs (rkin430GFP, expressed and purified as described before[4]), which include the first 430 amino acids of kinesin-1 fused to GFP and a polyhistidine tag at the tail domain. For low ionic strength measurements, the channels were rinsed with BRB10 before adding the motility solution. In BRB10, a five-fold lower kinesin concentration was sufficient to achieve the same number of motility events as observed in BRB80. Kinesin motility was imaged using Total Internal Reflection Fluorescence (TIRF) microscopy. The kinesin stepping assay were performed at a controlled temperature of 28 °C. The temperature was maintained using an objective heater.

Roadblock Experiments in BRB80 and BRB10 Buffers

Flow cells were prepared and coated with kinesin as described earlier. Alexa488- and Alexa647-labeled microtubules were diluted 10-fold in BRB80 motility solution, added to the flow cell, and incubated for 1 minute. After washing off excess microtubules, anti-AlexaFluor488 antibodies (Rabbit monoclonal IgG fraction, A11094, Invitrogen) were diluted in BRB80 motility solution and introduced into the flow cell. The anti-Alexa488 antibody solution was incubated for 2 minutes, and unbound antibodies were removed by washing the channel three times with three times the channel volume of BRB80 motility solution. Microtubule gliding was then imaged under a microscope for 5 minutes.

To assess the roadblock effect under low ionic strength conditions, in the same flow cell the buffer was exchanged with BRB10 motility solution by washing three times as before, and gliding microtubules were recorded for another 5 minutes. Roadblock experiments for different anti-Alexa488 antibody dilutions were performed using the same procedure. Each experimental condition was conducted using a single flow cell channel for both buffers to minimize variability.

For buffer-switching experiments with roadblocks, microtubules were incubated with a 1 nM anti-Alexa488 antibody solution in BRB80 motility buffer, and gliding was recorded as described above for both BRB80 and BRB10 buffers. The buffer in the flow cell was then switched back to BRB80 using three exchanges, and a final 5-minute imaging session was conducted. This approach ensured consistent conditions across measurements. The relative gliding speed was calculated as the ratio of the gliding speed of the test microtubule population (Alexa488-labeled microtubules) to that of the control population (Alexa647-labeled microtubules).

Roadblock Experiments in Modified BRB80 Buffers

Low ATP Buffer:

To match the gliding speeds of microtubules in BRB80 with those in low ionic strength conditions, we prepared a modified BRB80 motility buffer with reduced ATP concentration of 0.15 mM, while maintaining the total magnesium concentration at 3 mM, consistent with standard BRB80. This buffer was prepared by diluting a stock solution of 100 mM Mg-ATP in 100 mM $MgCl_2$, followed by mixing with the BRB80 motility solution. All other reagent concentrations remained unchanged. In this experiment, microtubules were first imaged in standard BRB80 buffer, followed by buffer exchange with the low ATP BRB80 buffer, and then with BRB10 motility buffer. Each condition was imaged for 5 minutes, with the roadblock effect tested at an anti-Alexa488 antibody concentration of 100 nM.

Low $MgCl_2$ and Low EGTA:

For this experiment, we used a modified BRB80 motility buffer in which magnesium and EGTA concentrations were adjusted to match those in BRB10 (0.25 mM $MgCl_2$, 0.25 mM EGTA). This modified buffer (80 mM PIPES, 0.25 mM EGTA, 0.25 mM $MgCl_2$) was supplemented with the same reagent concentrations as mentioned before to prepare the motility solution. The total $MgCl_2$ concentration in this buffer was 2.25 mM, instead of 3 mM in standard BRB80. The microtubules were imaged after a 2-minute incubation with 100 nM anti-Alexa488 antibody solution with standard BRB80, followed by the modified magnesium buffer, and finally in BRB10 motility solution. Roadblock effects were assessed using the same procedure, ensuring consistent imaging conditions across buffers.

BRB20 buffer

To test a broader range of ionic strength, we performed roadblock assay with 100 nM anti-Alexa488 antibody solution with an intermediate ionic strength buffer BRB20 buffer (20 mM PIPES, 0.25 mM EGTA, 0.25 mM $MgCl_2$) and compared the relative slowdown with standard BRB80 buffer and BRB10 buffer. The assay conditions were similar to low ATP and low $MgCl_2$, low EGTA buffer experiments. The microtubules were imaged after a 2-minute incubation with 100 nM anti-Alexa488 antibody solution with standard BRB80, BRB20 and BRB10 buffer.

Image Acquisition

Gliding motility assays were imaged using an upright fluorescence microscope (Nikon Eclipse Ni) equipped with a CFI S Plan Fluor ELWD 20X long-distance, wide-field, air objective and an ORCA-Fusion 14440 camera. Images were captured and processed with NIS-Elements software (Nikon). The microscope was fitted with a multiband filter (DAPI/FITC/Cy3/Cy5/Cy7 Penta-LED HC Filter, Analysentechnik) to visualize Alexa488- and Alexa647-labeled microtubules. The exposure time was set to 1 second and a timelapse video was recorded with an interval of 4 seconds between frames.
Single-molecule kinesin stepping assays were imaged using Total Internal Reflection Fluorescence (TIRF) microscopy on a Nikon Eclipse Ti2 microscope equipped with a perfect focus system, a 100×/1.49 NA oil apochromat TIRF objective, and a 1.5× optovar. Samples were illuminated with 488 nm and 647 nm lasers (100 mW each) from a Visitron laserbox channeled through an iLas2 ring TIRF module in single-angle mode. Fluorescent images were acquired using separate EMCCD cameras: an iXon Life EMCCD for the 488 nm channel and an iXon Ultra EMCCD for the 647 nm channel. Both cameras featured 1024×1024-pixel sensors, and pixel size was 87×87 nm. Images were acquired in streaming mode with 200 ms exposure (5.0 frames per second) and controlled using VisiView software.

Data Analysis and Statistical Methods

Microtubule gliding speeds were measured using an open-source MATLAB script[5] (AutoTipTrack), which tracks the microtubule ends in each frame with sub-pixel resolution. The algorithm calculates gliding speeds by determining the frame-to-frame velocities, based on the distance traveled along each path divided by the time between frames. The median and interquartile range was calculated from the frame-to-frame velocities. In the case of dual channel videos, the channels were first split using Fiji ImageJ software and then analyzed using AutoTipTrack algorithm.
The movement of kinesin molecules was analyzed with Fluorescence Image Evaluation Software for Tracking and Analysis[6] (FIESTA), which automates Gaussian fitting to extract X–Y position coordinates from fluorescence signals. Tracks were manually curated to remove errors before further analysis.
Results were plotted using Origin Pro 2024b and MATLAB (MathWorks 2018a). Linear regression was performed with Origin Pro 2024b. The overlay images were made using FIJI. Statistical significance for the gliding assay experiments was assessed using the one-tailed Wilcoxon rank-sum test with the ‘ranksum’ function in MATLAB. For the kinesin stepping assay, significance was evaluated using the two-sided student’s *t*-test conducted with the ‘ttest2’ function in MATLAB.

Supplementary Figures

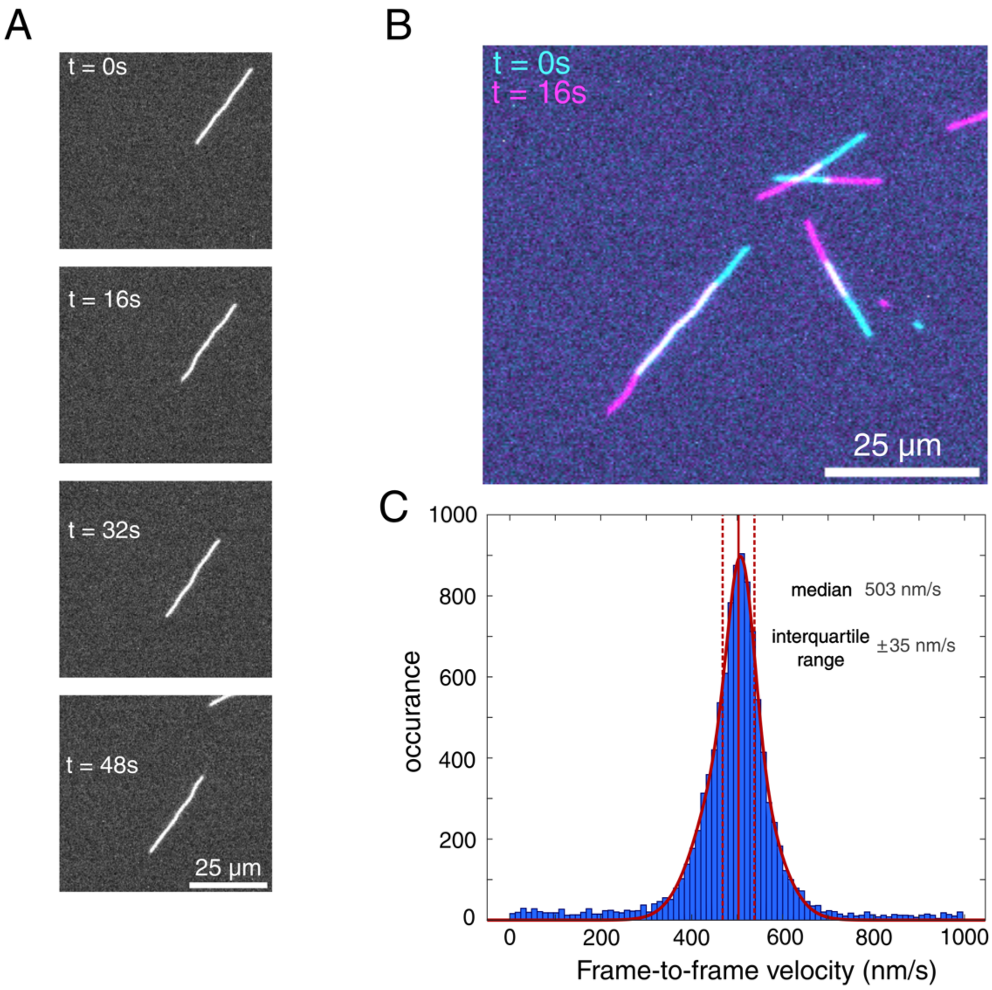

Figure S1: Tracking and Measurement of Microtubule Gliding Speeds. (A) Representative microscopy images showing a gliding microtubule at sequential time points. **(B)** Overlay image illustrating the position of the same microtubule at time points t=0s (cyan) and t=16s (magenta). Regions of overlap between the two positions are displayed in white. **(C)** Example calculation of microtubule gliding speed using an open-source MATLAB script (AutoTipTrack). Frame-to-frame velocities of the gliding microtubules were extracted and fitted to a Gaussian function (red curve). The median velocity (solid red line) and interquartile range (dotted red lines) were determined.

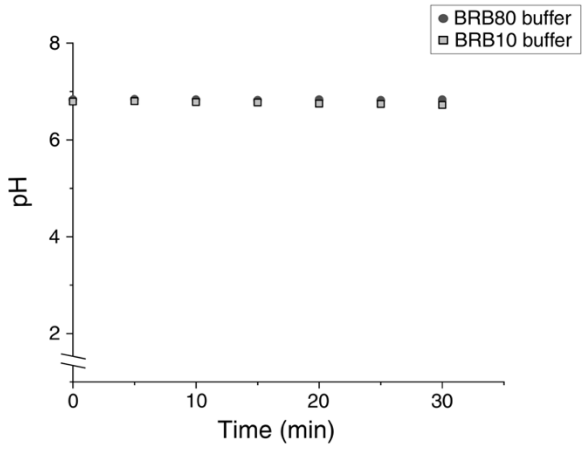

Figure S2: pH Stability of Standard BRB80 Motility Solution and Low Ionic Strength BRB10 Motility Buffer The pH stability of BRB80 (standard motility solution) and BRB10 (low ionic strength buffer) was monitored over time. The x-axis represents time in minutes, and the y-axis represents pH measurements.

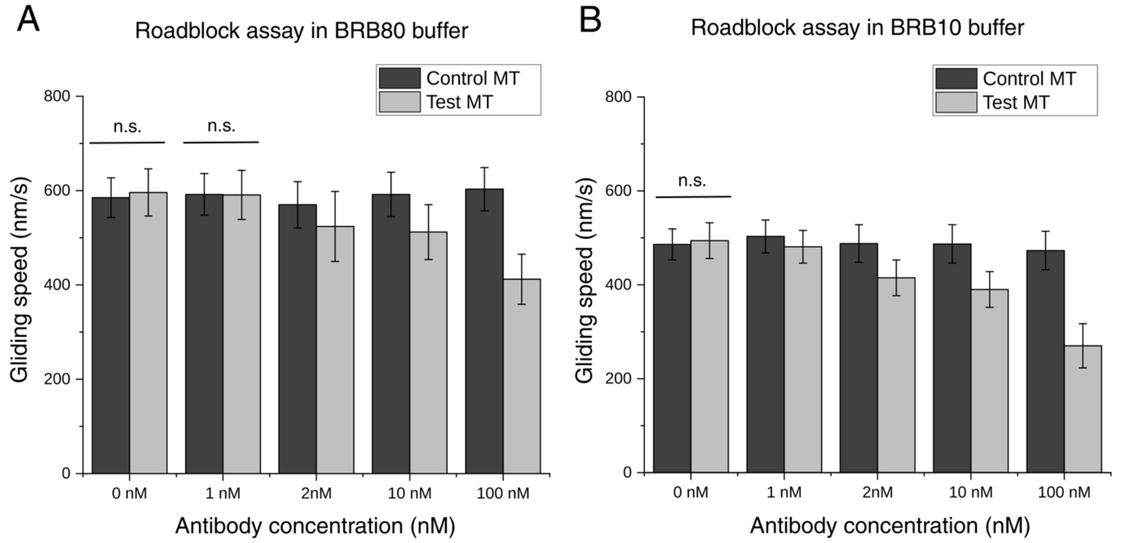

Figure S3: Comparison of the Effect of Low Ionic Strength Buffer (BRB10) on Roadblock Assay with Varying Anti-Alexa488 Antibody Concentrations. (A) Bar plot showing the gliding speeds of the control population (Alexa647-microtubules) and test population (Alexa488-microtubules) under standard BRB80 buffer conditions for different anti-Alexa488 antibody concentrations. Markers and error bars represent the median and interquartile range. The gliding speeds were significantly different on a 95% confidence level for antibody concentrations of 2 nM to 100 nM (Wilcoxon rank-sum test, $p < 0.001$) and not significantly different for 0 nM and 1 nM antibody concentration (n.s.). **(B)** Bar plot showing the gliding speeds of the control population (Alexa647-microtubules) and test population (Alexa488-microtubules) under low ionic strength BRB10 buffer conditions for different anti-Alexa488 antibody concentrations. Markers and error bars represent the median and interquartile range. The gliding speeds were significantly different on a 95% confidence level for antibody concentrations of 1 nM to 100 nM (Wilcoxon rank-sum test, $p < 0.001$) and not significantly different for 0 nM antibody concentration (n.s.).

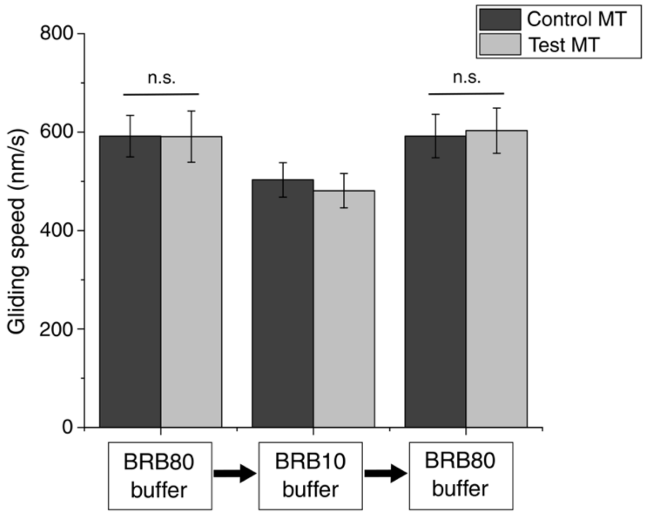

Figure S4: The Effect of Buffer Switching on Roadblock Assay. Bar plot showing the gliding speeds of the control population (Alexa647-microtubules) and test population (Alexa488-microtubules) for 100 nM anti-Alexa488 antibody concentration when buffers are alternated within the same channel between BRB80 and BRB10 buffers. Markers and error bars represent the median and interquartile range. The relative gliding speeds differed significantly under BRB10 buffer conditions (Wilcoxon rank-sum test, $p < 0.001$, 95% confidence level), whereas no significant difference was observed under BRB80 buffer conditions (n.s.).

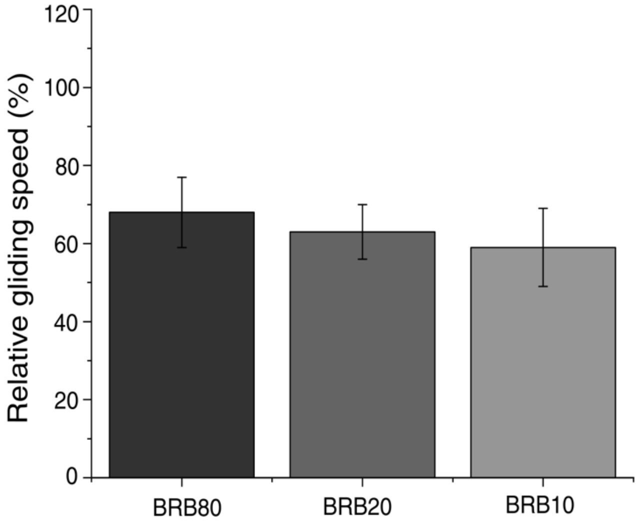

Figure S5: The Effect of Ionic strength on Roadblock Assay. Bar plot showing the relative gliding speeds (%) of microtubules in the presence of 100 nM anti-Alexa488 antibody concentration under varying ionic strengths. A general trend of increase in assay sensitivity with decrease in ionic strength was observed. Markers and error bars represent the median and interquartile range. The relative gliding speeds differed significantly under BRB80, BRB20 and BRB10 buffer conditions (Wilcoxon rank-sum test, $p < 0.001$, 95% confidence level)

REFERENCE(S)

(1) Pyrpassopoulos, S.; Gicking, A. M.; Zaniewski, T. M.; Hancock, W. O.; Ostap, E. M. KIF1A Is Kinetically Tuned to Be a Superengaging Motor under Hindering Loads. *Proc. Natl. Acad. Sci.* **2023**, *120* (2), e2216903120. https://doi.org/10.1073/pnas.2216903120.

(2) Castoldi, M.; Popov, A. V. Purification of Brain Tubulin through Two Cycles of Polymerization–Depolymerization in a High-Molarity Buffer. *Protein Expr. Purif.* **2003**, *32* (1), 83–88. https://doi.org/10.1016/S1046-5928(03)00218-3.

(3) Korten, T.; Chaudhuri, S.; Tavkin, E.; Braun, M.; Diez, S. Kinesin-1 Expressed in Insect Cells Improves Microtubule in Vitro Gliding Performance, Long-Term Stability and Guiding Efficiency in Nanostructures. *IEEE Trans. NanoBioscience* **2016**, *15* (1), 62–69. https://doi.org/10.1109/TNB.2016.2520832.

(4) Rogers, K. R.; Weiss, S.; Crevel, I.; Brophy, P. J.; Geeves, M.; Cross, R. KIF1D Is a Fast Non-processive Kinesin That Demonstrates Novel K-loop-dependent Mechanochemistry. *EMBO J.* **2001**. https://doi.org/10.1093/emboj/20.18.5101.

(5) Korten, T.; Tavkin, E.; Scharrel, L.; Kushwaha, V. S.; Diez, S. An Automated in Vitro Motility Assay for High-Throughput Studies of Molecular Motors. *Lab. Chip* **2018**, *18* (20), 3196–3206. https://doi.org/10.1039/C8LC00547H.

(6) Ruhnow, F.; Zwicker, D.; Diez, S. Tracking Single Particles and Elongated Filaments with Nanometer Precision. *Biophys. J.* **2011**, *100* (11), 2820–2828. https://doi.org/10.1016/j.bpj.2011.04.023.
